# Antioxidant Capacity of *Ocimum basilicum* L. and *Origanum vulgare* L. Extracts

**DOI:** 10.3390/molecules16097401

**Published:** 2011-08-30

**Authors:** Biljana Kaurinovic, Mira Popovic, Sanja Vlaisavljevic, Svetlana Trivic

**Affiliations:** Department of Chemistry, Biochemistry and Environmental Protection, Faculty of Sciences, Trg Dositeja Obradovica 3, Novi Sad 21000, Serbia

**Keywords:** free radicals, antioxidant capacity, *O. basilicum*, *O. vulgare*

## Abstract

The antioxidant properties of five different extracts (Et_2_O, CHCl_3_, EtOAc, *n*-BuOH, and H_2_O) of *Ocimum basilicum* L. and *Origanum vulgare* L. were studied. Antioxidant activity was assessed in six different model systems. Free radical scavenging capacity (RSC) was evaluated by measuring the scavenging capacity of extracts on DPPH, NO, O_2_^•^^−^ and OH radical, as well as on hydrogen peroxide (H_2_O_2_). In addition, the protective effects on lipid peroxidation in liposomes (LPx) were evaluated by TBA-assay using the Fe^2+^/ascorbate induction system. The amount of total phenolic compounds and content of total flavonoids was also determined. EtOAc, *n*-BuOH and H_2_O extracts of *O. basilicum* and *O. vulgare* expressed very strong scavenger activity. Furthermore, the mentioned extracts showed notable inhibition of LPx. On the other hand, Et_2_O and CHCl_3_ extracts showed much weaker effect in the neutralization of DPPH, NO and O_2_^•^^−^ radicals and the neutralization of H_2_O_2_. When examining the production of OH radicals and inhibition of LPx, the Et_2_O and CHCl_3_ extracts showed weak prooxidative properties. The observed differences in antioxidant activity could be partially explained by the levels of phenolics and flavonoids in the investigated *O. basilicum* and *O. vulgare* extracts.

## 1. Introduction

In the last three decades, especially in the developed countries of Europe and America, scientists have shown increasing interest in plant research. It is estimated that today about 60% of the total world population in treatment relies on herbs and natural products that are thus recognized as an important source of drugs [1]. Phytochemistry studies a huge variety of organic substances that have been discovered, and which accumulate in plants. Futhermore, phytochemistry is also defining the structure of these compounds, their biosynthesis, metabolism, their biosynthesis, metabolism, natural distribution and biological activities [2]. An important place among them is occupied by aromatic plants, whose aroma is associated with the presence of essential oils, complex mixtures of volatile compounds, dominated by mono- and sesquiterpenes. 

In addition to essential oils, aromatic plants are characterized by the presence of plant phenolic compounds, primarily coumarins and phenylpropanoids, that have been shown to possess multiple pharmacological activities. Investigations of these secondary biomolecules intensified when some commercial synthetic antioxidants were found to exhibit toxic, mutagenic and carcinogenic effects [3]. It was also found that excessive production of oxygen radicals in the body initiates the oxidation and degradation of polyunsaturated fatty acids. It is known that free radicals attack the highly unsaturated fatty acid membrane systems and induce lipid peroxidation, which is a key process in many pathological conditions and one of the reactions that cause oxidative stress. Particularly vulnerable are the biological membrane lipids in the spinal cord and brain, because they contain high levels of polyunsaturated fatty acids. Moreover, the brain contains significant amounts of transitional pro-oxidant metals and consumes a lot of oxygen. These features facilitate the formation of oxygen radicals involved in the processes of aging, Alzheimer’s and Parkinson’s disease, ischemic heart damage, arthritis, myocardial infarction, arteriosclerosis and cancer. Phenolic antioxidants “stop” free oxygen radicals and free radicals formed from the substrate by donating hydrogen atoms or electrons. Many plant species and aromatic plants have been tested because of their antioxidant and antiradical activity [4].

Basil (*Ocimum basilicum* L., Lamiaceae) is an aromatic herb that is used extensively to add a distinctive aroma and flavor to food. The leaves can be used fresh or dried for use as a spice. Essential oils extracted from fresh leaves and flowers can be used as aroma additives in foods, pharmaceuticals, and cosmetics [5]. Traditionally, basil has been used as a medicinal plant in the treatment of headaches, coughs, diarrhea, constipation, warts, worms, and kidney malfunction. Major aroma compounds from volatile extracts of basil present anti-oxidative activity [6]. Among the many studies to determine the antioxidant activities of basil, most have focused mainly on the antioxidant activities of crude extracts, using methanol, acetone, or water as a solvent [7,8].

The oregano (*Origanum vulgare* L., Lamiaceae) family, is widely known as possessing therapeutic properties (diaphoretic, carminative, antispasmodic, antiseptic, tonic), being used in the traditional medicine systems of many countries. It has been widely used in the agricultural, pharmaceutical and cosmetic industries as a culinary herb, flavoring substances in food products, alcoholic beverages and in perfumery for its spicy fragrance [9]. Regarding the nonvolatile components, the extracts of oregano have the most effective antioxidant activity among aromatic herbs [10]. Different groups of researchers [11,12] have studied oregano alcohol extracts. The antioxidant effect of the mentioned extracts is generally attributed to the presence of rosmarinic and caffeic acid [13]. 

Synthetic antioxidants are widely used in many foods to retard undesirable changes as a result of oxidation. The use of butylated hydroxyanisole (BHA) and butlylated hydroxytoluene (BHT) have been restricted in food because of their carcinogenic effects. Therefore, the search for new natural antioxidant sources has been greatly intensified. With regard to all of this, in the present study, chemical screens of five different extracts of basil (*Ocimum basilicum* L., Lamiaceae) and oregano (*Origanum vulgare* L., Lamiaceae) were performed. Total phenolics and flavonoids were quantified and the antioxidant activity of the extracts was determined *in vitro* via neutralization of 2,2-diphenyl-1-picrylhydrazyl (DPPH), nitric oxide (NO), superoxide anion (O_2_^•^^−^), hydroxyl (OH) radicals and hydrogen peroxide (H_2_O_2_), as well as by the inhibition of lipid peroxidation (LPx) in the Fe^2+^/ascorbate induction system.

## 2. Results and Discussion

### 2.1. Determination of Total Phenolic and Flavonoid Content

Results of the amount of total phenolic contents and content of total flavonoids in *O. basilicum* and *O. vulgare* extracts are given in Table 1.

**Table 1:** The amount of total phenolic contents and content of total flavonoids in *O. basilicum* and *O. vulgare* extracts.

Samples		Et_2_O	CHCl_3_	EtOAc	*n*-BuOH	H_2_O
*Ocimum*	Total phenolic content	4.86 ± 0.03	4.21 ± 0.01	9.76 ± 0.03	8.45 ± 0.02	11.88 ± 0.02
*basilicum*	Total flavonoids	13.21 ± 0.04	12.98 ± 0.03	23.12 ± 0.02	19.28 ± 0.06	26.42 ± 0.01
*Origanum*	Total phenolic content	5.02 ± 0.01	4.72 ± 0.01	14.13 ± 0.05	8.27 ± 0.02	10.29 ± 0.04
*vulgare*	Total flavonoids	9.37 ± 0.06	13.12 ± 0.05	28.54 ± 0.07	16.11 ± 0.04	25.31 ± 0.07

Total phenolic content is expressed in mg GAE/g d.e. ± S.D; Content of total flavonoids is expressed in μg RE/g d.e. ± S.D.

The amount of total phenolics in *O. basilicum* and *O. vulgare* extracts ranged from 4.21 ± 0.01 mg GAE/g d.e. (CHCl_3_ extract from *O. basilicum*) to 14.13 ± 0.05 mg GAE/g d.e. (EtOAc extract of *O. vulgare*). A significant amount of these compounds has also been observed in the H_2_O extracts of both examined plants (11.88 ± 0.02 mg GAE/g d.e. for *O. basilicum* and 10.29 ± 0.04 mg GAE/g d.e. in *O. vulgare*). Furthermore, a considerable total flavonoids content was determined in the H_2_O and EtOAc extracts of *O. basilicum* and *O. vulgare*. A little less total flavonoids was determined in the *n*-BuOH extracts, while the smallest quantity of these compounds was found in the Et_2_O and CHCl_3_ extracts. Differences in the amount of total phenolic and flavonoid content between extracts can be explained by the different number of secretory structures in various plant tissues [14]. In addition, it was found that duration of the extraction plays an important role because after 48 h all cell walls were destroyed and all plant material was present in very small particles. This could lead to adsorption of already extracted substances onto the particles, so that smaller amounts of extract would pass through filter paper, which would result in a smaller amount of dried extract after the longest period of extraction. Furthermore, the obtained results could be related to the protective role of phenolics, especially the flavonoid aglycones, in plants collected on the outskirts of big cities. One of the functions of these biomolecules in plants, which are produced in response to ecological stress factors like pollution, is to serve as UV-B filters [15]. More investigation is required to explain the enhanced production of phenolics in certain geographic areas [2,16].

### 2.2. *In Vitro* Experiments

The antioxidant activity of *O. basilicum* and *O.* *vulgare* extracts has been evaluated in a series of *in vitro* tests. The DPPH radical is one of the most commonly used substrates for fast evaluation of antioxidant activity because of its stability (in radical form) and the simplicity of the assay. In the DPPH assay, the ability of the investigated extracts to act as donors of hydrogen atoms or electrons in transformation of DPPH into its reduced form DPPH-H was investigated (Table 2).

**Table 2:** IC_50_ values (μg/mL) of the neutralization of DPPH radical with *O. basilicum* and *O. vulgare* extracts.

				IC_50_ (μg/mL)			
**Extract**	**Et_2_O**	**CHCl_3_**	**EtOAc**	***n*** **-BuOH**	**H_2_O**	**BHT**	**BHA**
*O. basilicum*	24.91	21.14	12.86	17.67	8.17	14.31	11.08
*O. vulgare*	23.83	19.89	14.11	12.91	11.24	14.31	11.08

All of the assessed extracts of *O. basilicum* were able to reduce the stable, purple-colored radical DPPH to the yellow-colored DPPH-H form with IC_50_ (50% of reduction) values as follows: 8.17 μg/mL for H_2_O, 12.86 μg/mL for EtOAc, 17.67 μg/mL for *n*-BuOH, 21.14 μg/mL for CHCl_3_, and 24.91 μg/mL for Et_2_O extract. Comparison of the DPPH scavenging activity of the investigated *O. basilicum* extracts with those expressed by BHT (14.31 μg/mL) showed that only the H_2_O and EtOAc extracts expressed stronger antioxidant effects. Comparison of DPPH activity of extracts *O. basilicum* with the activity exhibited by BHA (11.08 µg/mL) found that only the H_2_O extract showed stronger antioxidant activity. Examination of extracts of *O. vulgare* indicates that only the H_2_O and *n*-BuOH extracts showed stronger antioxidant effect than BHT, but neither of the extracts showed better antioxidant properties than BHA. Comparing with the DPPH test results of total flavonoids content in the extracts (Table 1), it could be concluded that only in case of the EtOAc and H_2_O extracts of *O. basilicum* and *O. vulgare* there is some correlation between the DPPH scavenger activity and content of flavonoids. This confirmed once again that the antioxidant capacity depends not only on the quantity, but also on the type of flavonoids present in the extracts. High activity of the H_2_O extract of *O. basilicum* and *O. vulgare* could be explained by the presence of caffeic acid derivatives in these extracts, which has two hydroxyl groups in the *ortho* position. Furthermore, it is obvious that, although the content of flavonoids in the EtOAc extract of *O. vulgare* was significantly higher than in the EtOAc extract of *O. basilicum*, their DPPH activity was equal. Preliminary 2D-TLC (Two Dimensional–Thin Layer Chromatography) analysis showed that the dominant flavonoid in the EtOAc extract of *O. basilicum* is a derivative of quercetin, while in the EtOAc extract of *O. vulgare* kaempferol glycosides were found [17]. It is known that quercetin shows higher antioxidant activity than kaempferol because of the OH groups present in position 3′ of its B ring (that includes a 3′,4′-dihydroxy group). In the case of *O. vulgare*, the *n*-BuOH extract showed a stronger ability to neutralize DPPH radicals than the EtOAc extract, although the content of flavonoids is higher in the latter. From this, it could be assumed that the *n*-BuOH extract contains more kaempferol monoglycoside while the EtOAc extract contains multiple kaempferol diglycosides. From the literature it is known that additional glycosylation reduces the antioxidant activity [18].

An extremely strong neutralization of NO radicals is expressed by a number of the tested extracts of *O. basilicum*, such as H_2_O (IC_50_ = 6.92 μg/mL) and EtOAc (7.71 μg/mL) (Table 3). Both extracts exhibited stronger antioxidant effects than BHT, and the effect of the H_2_O extract was closely to the action of BHA (6.31 μg/mL). In the case of extracts of *O. vulgare*, H_2_O (IC_50_ = 7.96 μg/mL) and EtOAc (9.33 μg/mL) showed the strongest activity, but this ability to neutralize of NO^•^ radicals is weaker than in the case of the relevant extracts of *O. basilicum*. The lowest antioxidant activity was expressed by the Et_2_O (IC_50_ = 21.11 μg/mL for *O. basilicum* and IC_50_ = 24.86 μg/mL for *O. vulgare*) and CHCl_3_(IC_50_ = 25.45 μg/mL for *O. basilicum* and IC_50_ = 27.24 μg/mL for *O. vulgare*) extracts of both plants.

**Table 3:** IC_50_ values (μg/mL) of the neutralization of NO radical with *O. basilicum* and *O. vulgare* extracts.

				IC_50_ (μg/mL)			
**Extract**	**Et_2_O**	**CHCl_3_**	**EtOAc**	***n*** **-BuOH**	**H_2_O**	**BHT**	**BHA**
*O. basilicum*	21.11	25.45	7.71	12.17	6.92	8.63	6.31
*O. vulgare*	24.86	27.24	9.33	14.92	7.96	8.63	6.31

Our results could be partly explained by a high content of total phenols and flavonoids (Table 1) in extracts of *O. basilicum* and *O. vulgare*, both free and sugar bound, due to the strong antioxidant activity of herbal products, according literature data, primarily related to the presence of different classes of phenolic compounds [19,20]. Differences between the activity of certain extracts of *O. basilicum* and *O. vulgare* are clearly visible and directly correlated with large variations in the chemical composition of the tested extracts. They also confirm earlier published results of very significant differences in the neutralization of peroxynitrite (ONOO^−^) that are directly correlated with manner of preparation and chemical composition of the tested extracts [21].

Superoxide anion radical generated by one-electron reduction of molecular oxygen or oxidation of one- electron reduction of hydrogen peroxide. A significant number of enzymatic reactions in biological systems result in the creation of this radical, and the highest amounts are produced in reactions of oxidases such as xanthine oxidase (XOD), aldehyde oxidase, but also reactions catalyzed by NADPH-cytochrome C reductase, NADPH-cytochrome P_450_reductase, *etc*. In the reaction with H_2_O_2_, superoxide anion radical produces hydroxyl ion (Haber-Weiss or Fenton reaction), while the reaction with nitrogen (I) oxide forme peroxynitrite anion (ONOO^−^) which may have greater toxicity the extracellular OH radical [22]. Table 4 shows the results of spectrophotometric measurements of the inhibition of superoxide anion radical by the *O. basilicum* and *O. vulgare* extracts, as well as commercial synthetic antioxidants (BHT and BHA).

**Table 4:** IC_50_ values (μg/mL) of the neutralization of O_2_^•^^−^ radical with *O. basilicum* and *O. vulgare* extracts.

				IC_50_ (μg/mL)			
**Extract**	**Et_2_O**	**CHCl_3_**	**EtOAc**	***n*** **-BuOH**	**H_2_O**	**BHT**	**BHA**
*O. basilicum*	17.21	16.84	10.73	14.62	10.61	10.46	8.41
*O. vulgare*	15.36	15.79	11.14	13.48	7.28	10.46	8.41

The data presented in Table 4 indicated that *O. basilicum* and *O. vulgare* extracts showed a strong effect on the inhibition of superoxide anion radical, even at very low applied concentrations. The strongest scavenger activity, stronger than that of commercial antioxidants, was displayed by the H_2_O extract from *O.* *vulgare* (IC_50_ = 7.28 μg/mL). The activity of the EtOAc and H_2_O extracts obtained from *O. basilicum* and the EtOAc extract from *O. vulgare* was approximately equal to that of the synthetic antioxidant BHT and slightly weaker than the activity of BHA. Relatively weaker scavenger activity on superoxide anion radical was shown by the Et_2_O, CHCl_3_ and *n*-BuOH extracts of *O. basilicum* and *O. vulgare*, although it should be noted that in this case the IC_50_ value was achieved at very low working concentrations. 

Although hydrogen peroxide is not a free-radical species, it is the source of the very toxic hydroxyl radical, especially in the presence of metal ions like iron or copper. Furthermore, H_2_O_2_ can cross membranes and may slowly oxidise a number of cell compounds. Thus, the elimination of H_2_O_2_, as well as the OH radical is very important for protection of pharmaceutical and food systems [23]. The neutralization of H_2_O_2_ by the examined extracts was measured spectrophotometrically (Table 5).

**Table 5:** IC_50_ values (μg/mL) of the neutralization of H_2_O_2_ radical with *O. basilicum* L. and *O. vulgare* extracts.

				IC_50_ (μg/mL)			
**Extract**	**Et_2_O**	**CHCl_3_**	**EtOAc**	***n*-BuOH**	**H_2_O**	**BHT**	**BHA**
*O. basilicum*	71.42	65.81	24.16	35.65	17.93	19.96	17.84
*O. vulgare*	68.17	62.13	39.24	51.15	26.58	19.96	17.84

Based on the results of the spectrophotometric measurement of the H_2_O_2_, in reference to reducing its concentration in solution, we can see that all the examined *O. basilicum* and *O. vulgare* extracts showed the ability to remove H_2_O_2_. Extremely high RSC, as well as in the case of neutralization of DPPH and NO radicals, was found in the H_2_O extract of *O. basilicum* (IC_50_ = 17.93 μg/mL). As opposed to the H_2_O extract of *O. basilicum*, other tested extracts exhibited much weaker antiradical effects. The closest “scavenger” activity among them was displayed by the Et_2_O extracts of *O. basilicum* (IC_50_ = 71.42 μg/mL) and *O. vulgare* (IC_50_ = 68.17 μg/mL) on the one hand, and the CHCl_3_ extracts of *O. basilicum* (IC_50_ = 65.81 μg/mL) and *O. vulgare* (IC_50_ = 62.13 μg/mL) on the other hand. Similar IC_50_ values of these extracts can be explained by the similarity in the content of certain classes of phenolic compounds. Significant differences in the process of neutralization of H_2_O_2_ were also found in the literature, and depend, as in the case of DPPH radicals, on the growth stage of the plants, plant materials and methods of preparation of plant extracts [24].

**Figure 1:**
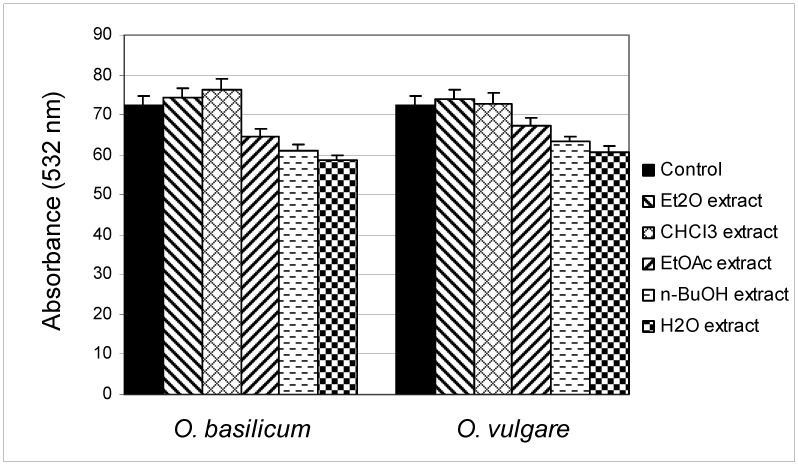
Inhibition of degradation of 2-deoxyribose by different extracts of *O. basilicum* and *O. vulgare* in the deoxyribose assay.

Hydroxyl radical (OH) is chemically the most reactive form of “activated oxygen”, which occurs during the reduction of molecular oxygen and it is the form most responsible for the cytotoxic effects of oxygen [25]. Due to its extreme reactivity, hydroxyl radicals react immediately with biomolecules and can effectively “attack” every molecule present in living cells such as sugars, amino acids, phospholipids, pyrimidine and purine bases and organic acids [26]. The OH radical scavenging capacity of *O. basilicum* and *O. vulgare* extracts was measured using the deoxyribose assay (Figure 1).

*O. basilicum* extracts exhibited different behavior with respect to the production of OH radicals. The EtOAc, *n*-BuOH and H_2_O extracts showed inhibition of OH radical, with the strongest OH radical production inhibition being expressed by the H_2_O extract (58.6 ± 1.2 nmol/mL) in comparison with the control (72.5 ± 2.1 nmol/mL). The Et_2_O and CHCl_3_ extracts have prooxidative effects, but not statistically significant. Similar behavior was found when examining the effects of *O.* *vulgare* extracts on the production of OH radicals—EtOAc, *n*-BuOH and H_2_O extracts expressed inhibition of OH radical. However, although this test is used as a valid method for determining the degree of inhibition of OH radicals, the relatively poor ability to neutralize OH radicals by some extracts also does not necessarily imply low antioxidant properties, because some recent research in model-systems indicates that the inhibition of deoxyribose did not seem to represent standard “scavengers” of OH radical like dimethylsulfoxide (DMSO). Therefore, it is assumed that degradation of deoxyribose in this test can be initiated by some other reactive radical species and transition metal ions [27]. 

**Figure 2:**
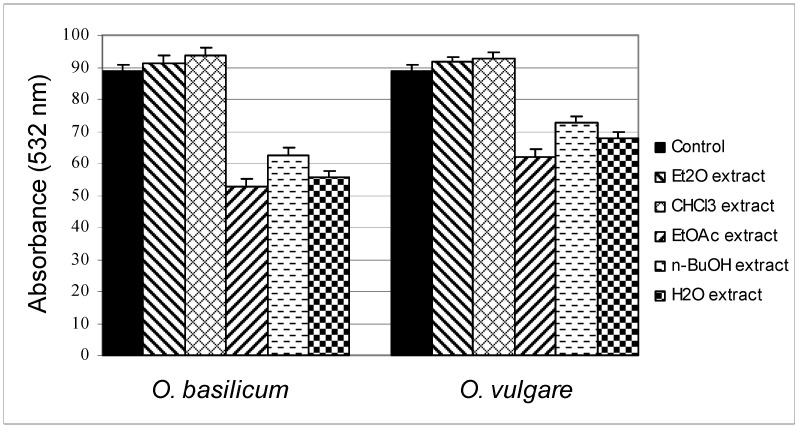
Inhibition of LP in Fe^2+^/ascorbate system of induction by different extracts of *O. basilicum* and *O. vulgare* in the TBA assay.

Since lipid peroxidation causes oxidative damage to cell membranes and all other systems that contain lipids [28], in any investigation of total antioxidative activity of extracts it is necessary to investigate their effects on lipid peroxidation. Some substrates (liposomes and linolenic acids) are used more frequently than others, mainly because of the simplicity of the methods involved. Also, due to their complex composition, examining the process of lipid peroxidation in fatty oils and liver homogenates makes research more difficult. For all these reasons, determining the impact of the tested extracts on lipid peroxidation process was performed on liposomes and the results of this activity are shown in Figure 2.

The data presented in Figure 2 show that the last three extracts of *O. basilicum* (EtOAc, *n*-BuOH and H_2_O) reduced the intensity of lipid peroxidation. The largest inhibitory activity was exhibited by the EtOAc extract. The high inhibitory effects of these three extracts can be related to the presence of the amount of total phenolic contents and content of total flavonoids in the extracts. It was established that flavonoids act as powerful scavengers of free radicals [29]. Different flavonoids inhibit LP *in vitro* and the most pronounced effect is exhibited by quercetin, whose presence is found in extracts of *O. basilicum* using 2D-TLC [30]. Similar behavior was found when examining the influence of extracts of *O. vulgare* on the intensity of lipid peroxidation. The first two extracts (Et_2_O and CHCl_3_) have prooxidative effects on the intensity of LPx (but not statistically significant), while the last three extracts reduced LPx intensity. The largest inhibitory activity, again, was exhibited by the EtOAc extract. If we compared the effect of the two EtOAc extracts with the amount of total phenolic contents and flavonoids (Table 1), it is understandable why the EtOAc extract of *O. vulgare* had the strongest inhibitory effect. However, in the extracts of *O. basilicum* the H_2_O extract had a higher content of total phenols and flavonoids than the EtOAc extract. From all this it can be assumed that the polarity of flavonoid components affects their ability to inhibit the LPx process. Specifically, in this test we used liposomes as a model system of biological membranes, and the least polar flavonoids present in the EtOAc extract might be able to better access the reaction sites and engage in the process of defense from the LPx, compared to more polar flavonoids found in the H_2_O extract.

## 3. Experimental

### 3.1. Chemicals

Thiobarbituric acid (TBA), gallic acid, xanthine, xanthine-oxidase, ethylenediaminetetraacetic acid (EDTA), 2,2-diphenyl-1-picrylhydrazyl (DPPH) and trichloroacetic acid were purchased from Sigma-Aldrich Chem (Steinheim, Germany). Folin-Ciolcateu reagent was provided by Fisher Scientific (Leicestershire, UK). 2-Deoxy-D-ribose was purchased from Aldrich. *N***-**(1-naphthyl)-ethylenediamine dihydrochloride (NEDA) was acquired from Merck (Darmstadt, Germany). Rutin, *tert*-butyl hydroxytoluene and *tert*-butyl-4-hydroxyanisole were obtained from Fluka AG (Buchs, Switzerland). The commercial preparation of liposomes “PRO-LIPO S” was purchased from Lucas-Meyer (Hamburg, Germany). All chemicals used were of analytical grade.

### 3.2. General

Aerial parts of cultivated flowering plants of basil (*Ocimum basilicum* L.) and oregano (*Origanum vulgare* L.) were collected in July of 2008 in Vojvodina Province, Republic of Serbia. The plant materials (*O. basilicum* and * O. vulgare*) were dried in air and ground in a mixer. A portion of the finely powdered material (200 g) was extracted three times with 70% methanol (MeOH, 4 L) during a 24-h period. After removal of MeOH under reduced pressure, the aqueous phase was successively extracted with four solvents of increasing polarity, namely ether (Et_2_O), chloroform (CHCl_3_), ethylacetate (EtOAc) and *n*-butanol (*n*-BuOH). The extraction was carried out until a colourless extract was obtained. The residue was the aqueous extract. All five extracts (Et_2_O, CHCl_3_, EtOAc, *n*-BuOH, and H_2_O) were evaporated to dryness and then dissolved in 50% ethanol to make 10% (w/v) solutions. These solutions, either as such or in diluted state, were used in subsequent experiments.

### 3.3. Determination of Total Phenolic and Flavonoid Content

The amount of total phenolic contents in the extracts was determined spectrophotometrically with the Folin-Ciocalteu (FC) reagent using the method of Fukumoto and Mazza [31] with small modifications [32]. The reaction mixture contained 1.0% dilution of examined extracts (100 µL), freshly prepared 0.2 M FC reagent (2.5 mL) and 10% sodium carbonate solution (2 mL). The mixture was incubated in the dark at room temperature for 1 hour to complete the reaction. The absorbance of the resulting solution was measured at 760 nm on a UV/VIS spectrophotometer (CECIL CE2021) using distilled water as the blank. The concentration of total phenolic contents was expressed in mg gallic acid equivalents (GAE) per g dried extract (d.e.), using a standard curve of gallic acid (0.1-2.0 µg/mL). All measurements were replicated five times.

Total flavonoid content in the extracts was determined spectrophotometrically according to Jia *et al*. [33], using a method based on the formation of a flavonoid-aluminium complex with an absorbance maximum at 430 nm. The examined extracts (1 mL) were mixed with 2% AlCl_3_× 6H_2_O (0.5 mL). After incubation at room temperature for 30 min, the absorbance of the reaction mixtures was measured. The blank sample was a 1:1 mixture of the examined extracts and distilled water. Flavonoid content was expresed in µg rutin equivalent (RE) per g dried extract by using a standard curve of rutin (concentration range 0.5–6.0 µg/mL). All measurements were replicated five times.

### 3.4. *In Vitro* Experiments

The DPPH assay was performed as described previously [34,35], following the transformation of the DPPH radical to its reduced, neutral form (DPPH-H). The samples of all extracts of *O. basilicum* and *O. vulgare* (from 2.50 to 50.00 μg/mL) were mixed with 90 μM DPPH^•^ solution (1 mL) and made up with 95% MeOH to a final volume of 4 mL. The absorbance of the resulting solutions was recorded spectrophotometrically at 515 nm after 1 h at room temperature, against the blank (with the same chemicals, except for the sample). The same procedure was repeated with *tert*-butylhydroxytoluene (BHT) and *tert*-butyl-4-hydroxyanisole (BHA) as a positive control. For each sample five replicates were recorded. 

Production of NO radicals was determined spectrophotometrically. NO radical generated from sodium-nitropruside (SNP) reacts with oxygen in water solution at a physiological pH to give nitrite ions. Concentration of nitrite anions was determined using the Griess reagent [36,37]. At room temperature nitrite ions react with the Griess reagent and form a purple complex. The samples of all *O. basilicum* and *O. vulgare* extracts were investigated in concentrations of 2.50–50.00 μg/mL. The intensity of color, which is the function of the nitrite concentrations, was measured spectrophotometrically (λ = 546 nm). The absorbance of the resulting solutions and the blank (with the same chemicals, except for the sample) were recorded. For each sample, five replicates were recorded.

Superoxide anion radicals were generated in the system xanthine/xanthine-oxidase, and the quantity of O_2_^•^^−^ was determined by the nitrite method with modifications [38]. All solutions and reagents were freshly prepared by dissolution in 0.05 M KH_2_PO_4_–K_2_HPO_4_ phosphate buffer (pH 7.4). The samples of all extracts of *O. basilicum* and *O. vulgare* were investigated in different concentrations (from 2.50 to 50.00 μg/mL). The absorbance of the resulting solutions was measured spectrophotometrically at 550 nm after 30 min at room temperature, against the blank (with the same chemicals, except for the xanthine-oxidase). The same procedure was repeated with BHT as a positive control. For each sample five replicates were recorded. 

Scavenging activity on H_2_O_2_ was carried out according to the method of Ruch *et al*. [39]. A solution of H_2_O_2_ (40 mM) was freshly prepared in 0.05 M KH_2_PO_4_–K_2_HPO_4_ phosphate buffer (pH 7.4). The samples (from 2.50 to 50.00 μg/mL) were mixed with phosphate buffer (3.4 mL) and 40 mM H_2_O_2_ (0.6 mL). The absorbance of the resulting solutions and the blank (4.0 mL phosphate buffer) was detected spectrophotometrically at 230 nm. The percentage of RSC for each radical and H_2_O_2_ was calculated using the following equation:

RSC(%) = 100 × (*A*_blank_ − *A*_sample_ ⁄ *A*_blank_)

From the obtained RSC values, the IC_50_ values, which represented the concentrations of the examined extracts that caused 50% neutralization, were determined by linear regression analysis.

The scavenging capacity of *O. basilicum* and *O. vulgare* extracts for hydroxyl radicals was determined by monitoring the chemical degradation of 2-deoxy-D-ribose [40]. The reaction was initiated by hydroxyl radicals obtained in Fenton’s reaction [41], which yields products that react with thiobarbituric acid (TBA test). The obtained products, among which malondialdehyde (MDA) is the most important, are determined by a spectrophotometric method at 532 nm. The absorbance of the resulting solutions and the blank (with same chemicals, except sample) was recorded. For the experiment, one concentration of extracts of *O. basilicum* and *O. vulgare* was prepared (10% solution). Five replicates were performed for each sample. 

The extent of LP was determined by measuring the color of the adduct produced in the reaction between 2-thiobarbituric acid (TBA) and malondialdehyde (MDA), as an oxidation product in the peroxidation of membrane lipids, by the TBA assay [42,43,44]. The commercial preparation of liposomes ‘PRO-LIPO S’ (Lucas-Meyer) pH = 5–7 was used as a model system of biological membranes. The liposomes, 225–250 nm in diameter, were obtained by dissolving the commercial preparation in demineralized water (1:10), in an ultrasonic bath. For the experiment, one concentration of extracts of *O. basilicum* and *O. vulgare* was prepared (10% solution). Five replicates were performed for each sample.

## 4. Conclusions

The measurement of antioxidant activity was used as a method for the evaluation of *O. basilicum* and *O. vulgare* extracts. The comparison of antioxidant activities of the investigated extracts showed variable effects that depended on the examined extracts and the model system used for investigation. Generally, the EtOAc, *n*-BuOH and H_2_O extracts of both plants possessed a strong antioxidant activity. In most of the tested models (reduction of DPPH, NO and OH radical and neutralization of H_2_O_2_), the H_2_O extract from *O. basilicum* expressed the strongest antioxidant activity. Only in the case of neutralization of O_2_^•^^−^ radical, the H_2_O extract from *O. vulgare* showed the best inhibitory properties. The results for the H_2_O extract of *O. basilicum* are logical, as it was found that this extract contains the highest amount of total phenolic contents and flavonoids. Furthermore, the best inhibitory effect of the EtOAc extracts from both plants on LPx indicates that the polarity of flavonoids affects their ability to inhibit LPx.
